# Determinants of cost of routine immunization programme in India

**DOI:** 10.1016/j.vaccine.2018.05.006

**Published:** 2018-06-18

**Authors:** Susmita Chatterjee, Arpita Ghosh, Palash Das, Nicolas A. Menzies, Ramanan Laxminarayan

**Affiliations:** a Public Health Foundation of India, Gurgaon, Haryana, India; b Department of Global Health and Population and Center for Health Decision Science, Harvard T.H. Chan School of Public Health, Boston, MA, USA; c Centre for Disease Dynamics, Economics & Policy, Washington, DC, USA; d Princeton Environmental Institute, Princeton University, Princeton, NJ, USA

**Keywords:** Cost, Determinant, Routine immunization, India

## Abstract

The costs of delivering routine immunization services in India vary widely across facilities, districts and states. Understanding the factors influencing this cost variation could help predict future immunization costs and suggest approaches for improving the efficiency of service provision.

We examined determinants of facility cost for immunization services based on a nationally representative sample of sub-centres and primary health centres (99 and 89 facilities, respectively) by regressing logged total facility costs, both including and excluding vaccine cost, against several explanatory variables. We used a multi-level regression model to account for the multi-stage sampling design, including state- and district-level random effects.

We found that facility costs were significantly associated with total doses administered, type of facility, salary of the main vaccinator, number of immunization sessions, and the distance of the facility from the nearest cold chain point.

Use of pentavalent vaccine by the state was an important determinant of total facility cost including vaccine cost. India is introducing several new vaccines including some supported by Gavi. Therefore, the government will have to ensure that additional resources will be made available after the support from Gavi ceases.

## 1 Introduction

The costs of delivering routine immunization services vary widely across facilities within countries and across countries [1], [2], [3], [4]. Understanding the reasons for such variation can provide insights into site operations and help improve programme efficiency. Recently, under the EPI Costing and Financing (EPIC) project, a few studies have investigated the cost drivers of routine immunization programmes [5], [6]; however, there is clearly a dearth of such evidence in large countries like India. Because immunization programmes differ across countries based on distribution of health care services, population characteristics, and vaccine schedules, country-level information on costs and cost determinants is important.

India’s national immunization programme was introduced in 1978 following the success of smallpox eradication [7]. The programme is the largest in the world and covers a birth cohort of 26 million infants for eight vaccine-preventable diseases: diphtheria, whooping cough, tetanus, poliomyelitis, tuberculosis, measles, hepatitis B, and *Haemophilus influenzae* type B (Hib) (which causes pneumonia and meningitis). The programme also provides vaccination for Japanese encephalitis in areas affected by the disease. Recently, a vaccine against rotavirus has been introduced in nine states, and pneumococcal vaccine has been introduced in a cohort of three states with a plan to rapidly scale up in other cohorts or states.

The total expenditure for India’s immunization programme as reported in the comprehensive multi-year plan for immunization (cMYP), was US$718 million in 2012–13 [8], and a recent study on routine immunization costs showed substantial variation in unit costs across facilities, districts and states [9]. During 2013–14, the weighted average state-level cost per dose delivered varied from US$1.31 to US$2.79 including the vaccine cost, while the cost per child vaccinated with the third dose of diphtheria, pertussis, tetanus (DPT) vaccine (a proxy for full immunization) varied from US$19.11to US$33.13 including the vaccine cost. In this study, we examine the factors underlying these cost variations and suggest approaches to improving the efficiency of service provision.

## 2 Methods

As part of the India immunization costing study, data on immunization service costs were collected from 255 government health facilities of different types across seven states, using a multi-stage cluster sampling design [9]. India’s 29 states were stratified into six levels of development based on indicators such as female literacy rate, full immunization coverage rate, infant mortality rate, and per capita income. The states were further classified into six regions – north, northeast, east, central, south and west. To ensure a nationally representative sample, the study used stratified purposeful sampling to select seven states representing all six levels of development and all six regions. Study states included Punjab (north); Meghalaya (northeast); Bihar and West Bengal (east); Uttar Pradesh (central); Kerala (south); and Gujarat (west). Although Bihar and Uttar Pradesh were at the same level of development, both were selected as they have high priority for improving immunization coverage.

The costing study had calculated the number of health facilities that would be required to estimate the mean cost per fully vaccinated child within a margin of error of US$3 with a 95% confidence interval in each of the selected states, after accounting for the multi-stage cluster sampling design. In the calculation, the standard deviation of the cost per fully immunized child was assumed to be US$8 or US$10, depending on the state under consideration. The two-stage cluster sampling design was accounted for by multiplying the sample size obtained under simple random sampling by the design effect of 1.2.

**Fig. 1:**
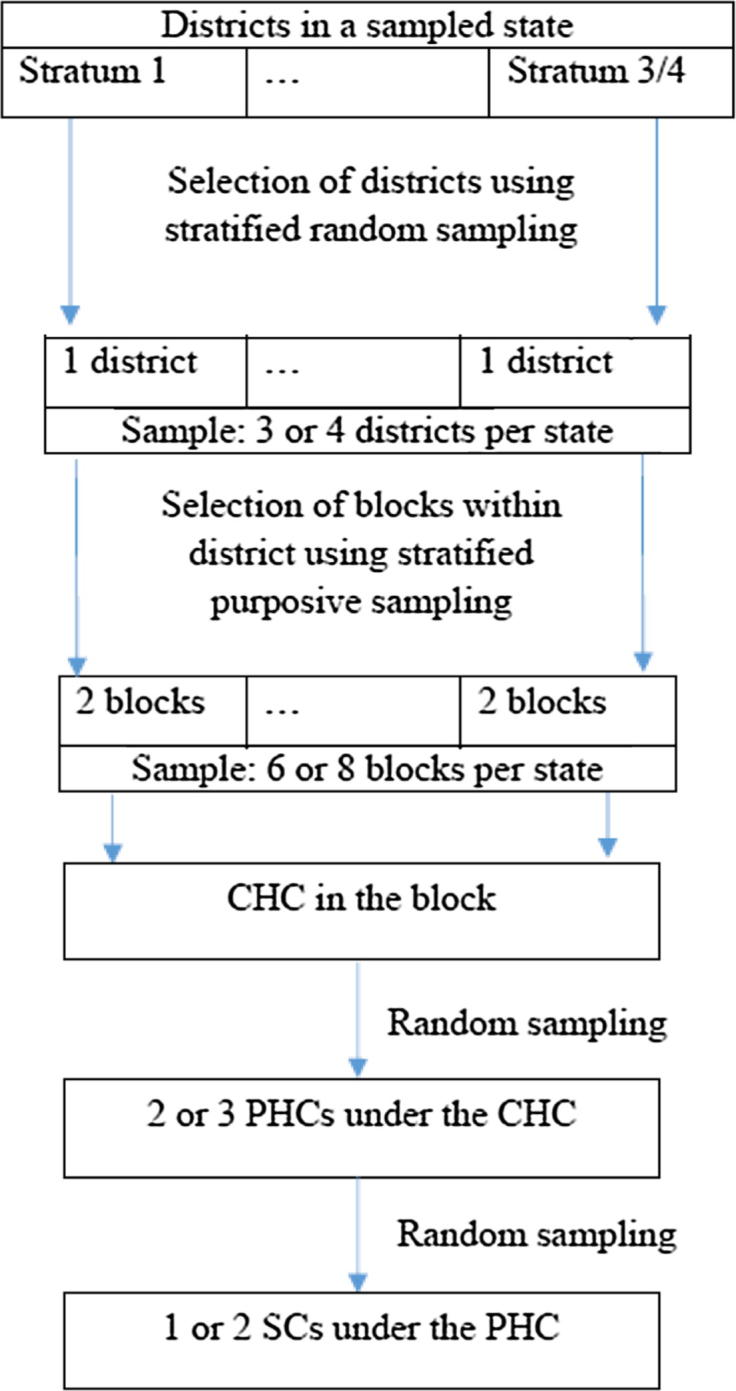
Flow chart describing sampling strategy within a selected state: same flow chart is applicable for all seven states. *Note:* CHC: community health centre; PHC: primary health centre; SC: sub-centre.

Because of time and resource constraints, the researchers reduced the number of health facilities in Uttar Pradesh and Bihar. The study comprised 24 health facilities each from Uttar Pradesh and Bihar; 48 health facilities each from Gujarat, Punjab and West Bengal; 33 from Kerala; and 30 from Meghalaya, resulting in a total of 255 facilities across seven states [9]. To ensure representativeness at the state level, the study used a stratified sampling design to select health facilities in each state. Districts in the selected states were divided into three or four strata based on the scores obtained from four district-level indicators: number of children aged 0–6 years, proportion of households in rural areas, proportion of children aged 0–6 years receiving full immunization, and number of health facilities per 1000 children. One district from each stratum was randomly selected using a computer application that employs random number generator. In each district, two blocks (sub-districts) were selected using purposive sampling based on two indicators: (1) the percentage of scheduled caste or scheduled tribe members in the population; and (2) the female literacy rate. The blocks were selected to cover the lower and upper extremes of these indicators. Each block typically has one community health centre (CHC),^2^ which was selected for inclusion in this study. In addition, two or three primary health centres (PHCs) associated with the CHC, as well as one or two sub-centres (SCs) under the selected PHCs were randomly selected. The final sample comprised 255 health facilities of four different types: 44 CHCs, 89 PHCs, 99 SCs, and 23 post-partum (PP) units at the district hospitals of the selected districts. A flow chart (Fig. 1) summarizes the sampling strategy followed within a selected state.

Economic costs of routine immunization were estimated from a government provider perspective and based on an approach that adapted a standardized method used for immunization costing studies (Common Approach) [11]. Total facility cost was the sum of personnel, capital costs, and all other recurrent expenses. Personnel costs were estimated based on salaries and allowances for various categories of staff involved in immunization and the time staff spent on activities related to immunization, which included conducting immunization sessions, transporting vaccines, keeping records, maintaining cold chains, monitoring and supervision, preparing microplans, and attending trainings and meetings. Capital costs included the annualized discounted value of cold chain equipment, vehicles and buildings. Costs for shared vehicles were based on the number of days a vehicle was used for immunization activities, while building costs were derived from the number of days a building was used for immunizations and the proportion of the building used. Other recurrent expenses were costs of vaccines and supplies, expenditures on trainings, meetings, vaccine transport, waste management, cold chain maintenance, printing, traveling to immunization sessions, and incentives, along with overhead expenses (e.g., for electricity and water). Overhead expenses were distributed to immunization and cold chain rooms based on share of space used for immunization.

Trained data collection teams visited each facility from October 2014 to October 2015. Data were gathered from financial reports, monthly immunization reports, immunization registers showing the total vaccines administered, and vaccine stock and issue registers. To determine person-time spent on immunization activities, the team interviewed staff involved in these activities. Data were also collected on possible determinants of immunization costs, including number of sessions and distance from the nearest cold chain point. Wastage rates for each vaccine at the vaccinator level were calculated using doses used and doses administered [9]. Total costs were estimated for one fiscal year, April 2013 to March 2014, and results were reported in 2013 US dollars. Throughout the paper, we used an exchange rate of US$1 = INR 58, the average exchange rate during 2013.

To understand the determinants of cost, in addition to primary data collection, we extracted block-level sociodemographic data from the 2011 census of India (the latest census data available). We collected data on variables including proportion of females who are literate, proportion of households belonging to scheduled castes or scheduled tribes, and proportion of households located in rural areas. Studies conducted in India had shown that immunization coverage is affected by sociodemographic characteristics such as literacy, caste, location and wealth index. Complete vaccination of a child is more likely if the mother is literate and the household belongs to a higher wealth quintile [12], [13], [14]. To estimate wealth, we extracted variables related to availability of facilities and asset possession of households and constructed a block-level wealth index using principal component analysis (PCA). The variables included the proportion of households in a given block reporting each of a wide range of amenities (tap water, electricity, a latrine, an improved latrine, non-use of solid fuel, banking services, a radio, a television, a computer, a phone, a two-wheel vehicle, a four-wheel vehicle). After deriving the principal components using PCA, we found that the first principal component explained 64% of the total variability. We therefore used the first principal component as the block-level wealth index. Based on the wealth index, we categorized the study blocks into quintiles: poorest, poorer, middle, richer, and richest.

### 2.1 Cost determinants model

We examined determinants of facility cost for immunization services provided by the SCs and PHCs by regressing logged total facility costs against several explanatory variables. For the main analysis, we considered total facility cost excluding vaccine cost as the outcome variable, as vaccine prices are fixed nationally and not affected by health facility performance. We adopted a multilevel model to account for the complex sample design with state- and district-level random effects, and we examined the relationship between facility-level total cost and explanatory variables at the facility, block, district and state levels. We used *MLwiN* (version 2.32) for fitting the multilevel models [15].

Our three-level regression model is

$$y_{\mathrm{ijk}}=\beta_{0}+\beta X_{\mathrm{ijk}}+f_{k}+v_{\mathrm{jk}}+{∊}_{\mathrm{ijk}}$$

where the outcome variable $y_{\mathrm{ijk}}$ is the logged total immunization cost excluding the vaccine cost for the *i*^th^ health facility in the *j*^th^ district in state *k*. The error term ${∊}_{\mathrm{ijk}}$ is normally distributed with mean 0 and variance $\sigma^{2}$. $X_{\mathrm{ijk}}$ represents the facility-, block-, district- and state-level variables. $f_{k}$ and $v_{\mathrm{jk}}$ are the random intercepts attributable to states and districts, respectively. The state- and district-level random intercepts are normally distributed with means 0 variances $\sigma_{f}^{2}$ and $\sigma_{v}^{2}$, respectively, and are independent of one another and of the error term.

We fitted a series of regression models starting with the null model without any fixed effects and only random components, and then we added ‘quantity’ (log doses administered) as the explanatory variable. The successive models had an increasing number of explanatory variables, including the health system level of the facility (health facility type: SC or PHC); other facility characteristics related to immunization activities (log distance from the nearest cold chain point and log number of immunization sessions); a proxy price measure based on the average salary of the main vaccinator; and, as a crude quality measure, the logged ratio of third doses of DPT vaccine to total doses administered. State-level per capita income in 2013–14 was considered as a crude index of price differences among states [16] and was included as a state-level covariate.

The selection of these explanatory variables was motivated by an earlier study of immunization delivery cost determinants in six countries (Benin, Ghana, Honduras, Moldova, Uganda and Zambia), which reported relationships between many of these variables and total facility cost [17]. As India immunization costing study adopted the same costing methodology used in these six countries (Common Approach), we included similar explanatory variables in the model.

The number of health facilities per 1000 children and the proportion of institutional deliveries in the district were used as proxies for availability of health services at the district level. To account for variations in sociodemographic and economic characteristics across the sampled blocks, we included several block-level variables — proportion of females who are literate, proportion of households belonging to a scheduled caste or scheduled tribe, proportion of households that are rural, and, lastly, the block’s wealth quintile. Because the regressions used logged costs, the regression coefficients are difficult to interpret directly. We therefore calculated first differences to demonstrate the implications of the regression results. We used simulation to estimate first differences and 95% confidence intervals (CI) for the predictors that were found to be significantly associated with the total cost. For continuous predictors, first differences were calculated to represent the difference between the 25th and 75th percentiles for each predictor.

Descriptive statistics of the explanatory variables are presented in Table 1.

**Table 1:** Descriptive statistics of explanatory variables.

	Bihar	Gujarat	Kerala	Meghalaya	Punjab	Uttar Pradesh	West Bengal
*Sub-centre*							
Number of facilities	9	20	12	9	20	10	19
Total facility cost with vaccine, median (IQR) (US$)	2966 (764)	2776 (961)	1468 (318)	1749 (817)	2820 (655)	2628 (426)	2249 (63 1)
Total facility cost without vaccine, median (IQR) (US$)	2271 (831)	1451 (477)	1346 (301)	1581 (888)	2570 (658)	2272 (399)	1987 (540)
Doses administered, median (IQR)	2338 (582)	1372 (744)	207 (56)	1340 (461)	1368 (370)	1748 (890)	1228 (513)
Main vaccinator's salary, median (IQR) (US$)	5627 (3694)	4567 (2177)	5087 (2221)	4450 (1463)	7144 (1433)	7972 (2617)	6319 (1195)
Distance from nearest cold chain point, median (IQR) (km)	8 (8)	10 (9)	3 (1)	10 (6)	9 (10)	13 (8)	9 (8)
Number of immunization sessions, median (IQR)	111 (26)	48 (17)	16 (12)	81 (29)	48 (14)	77 (26)	38 (10)
Proportion of DPT3 doses to total doses, average (SD)	0.07 (0.01)	0.09 (0.01)	0.11 (0.01)	0.08 (0.01)	0.07 (0.01)	0.08 (0.02)	0.07 (0.01)

*Primary Health Centre*							
Number of facilities	7	18	12	13	16	6	17
Total facility cost with vaccine, median (IQR) (US$)	3022 (3364)	6923 (2940)	7194 (1914)	6328 (950)	3850 (2399)	4399 (585)	3882 (1584)
Total facility cost without vaccine, median (IQR) (US$)	2731 (3165)	5490 (2478)	6661 (1488)	6100 (1112)	3590 (2348)	3970 (612)	3539 (1587)
Doses administered, median (IQR)	2438 (2027)	1280 (1135)	896 (794)	2485 (2074)	1388 (545)	2509 (539)	1671 (812)
Main vaccinator's salary, median (IQR) (US$)	6376 (2020)	4637 (2363)	5476 (1567)	5608 (1283)	7120 (2822)	8237 (751)	6586 (2198)
Distance from nearest cold chain point, median (IQR) (km)	15 (5)	30 (18)	7 (9)	44 (18)	12 (11)	12 (6)	10 (8)
Number of immunization sessions, median (IQR)	106 (22)	64 (33)	52 (9)	136 (74)	48 (15)	79 (25)	36 (17)
Proportion of DPT3 doses to total doses, average (SD)	0.07 (0.01)	0.09 (0.01)	0.12 (0.02)	0.08 (0.01)	0.07 (0.01)	0.07 (0.01)	0.07 (0.01)

*State, district and sub-district level variables*							
Pentavalent vaccine administered in study states	No	Yes	Yes	No	No	No	No
Per capita income of study states, 2013–14 (US$)	538	1842	1790	1061	1597	625	1208
Health facilities per 1000 children in study districts (average)	0.58	1.52	2.04	0.93	1.82	0.75	1.07
Percentage of institutional delivery in districts (average)	66	89	100	50	91	72	78
Female literacy rate in sub-districts, median (IQR)	44 (6)	60 (14)	87 (4)	58 (7)	56 (14)	49 (5)	56 (15)
Percentage of schedule caste/schedule tribe population in sub-districts, median (IQR)	18 (3)	13 (60)	9 (9)	97 (5)	31 (11)	16 (5)	27 (39)
Percentage of rural population in sub-districts, median (IQR)	76 (25)	84 (32)	54 (54)	100 (13)	71 (23)	84 (20)	88 (21)
Proportion of sub-districts in lowest wealth quintile	0.33	0.25	0.00	0.50	0.00	0.00	0.37

IQR = interquartile range; SD = standard deviation.

We decided against including CHCs and PP units in the regression. CHC operations differed substantially across states. For example, CHCs were the immunization planning units at the sub-district level for all study states except Gujarat. PHCs were the planning units in Gujarat, and CHCs there had very limited immunization activities. Hence, we dropped Gujarat CHCs, which reduced our sample size for a separate regression for CHCs. We did not combine PP units with SCs and PHCs because of the PP units’ different mode of operation. Hence, our regression results were based on combining data across 99 SCs and 89 PHCs.

## 3 Sensitivity analyses

We ran a separate regression for total facility costs including vaccine cost. In this model, in addition to state-level per capita income, we considered the use of pentavalent vaccine in a state as another predictor. Pentavalent vaccine was introduced in a phased manner in India. During the study period, two sampled states (Gujarat and Kerala) used pentavalent vaccine, while the other five states used DPT and hepatitis B vaccines. Use of pentavalent vaccine in a state probably contributed to cost variations across states and was considered as a predictor (Appendix Table S1).

In the main analysis, we conducted pooled regression analyses combining data from both SCs and PHCs in a single regression model. It is possible that the effects of different cost determinants vary by facility type, and as a robustness check we fitted separate regressions for SCs and PHCs. Although the effect size for doses administered on total facility cost was greater for SCs than for PHCs, most of estimated regression coefficients from these separate regressions were similar in magnitude and in the same direction (Appendix Table S2). We decided against running separate regressions for CHCs and PP units for reasons explained earlier.

## 4 Results

We present the regression coefficients from the final regression, including all explanatory variables, in Table 2. We found that total doses administered, type of facility (SC or PHC), salary of the main vaccinator and the number of immunization sessions were significantly associated with total facility cost excluding vaccine cost. On calculating first differences, we found that for 1 interquartile range increase in number of doses (1032 doses more), number of immunization sessions (42 sessions more) and salary of the main vaccinator (US$2228 higher), the average cost increased by US$239 (95% CI = US$46 to US$453), US$1062 (95% CI = US$713 to US$1439) and US$732 (95% CI = US$501 to US$982), respectively. The cost at sub-centres was found to be significantly lower compared to the cost at primary health centres by US$1934 (95% CI = US$1573 to US$2332).

**Table 2:** Results of the regression analysis of total facility cost excluding vaccine cost, for primary health centres and sub-centres.

Variable category	Variables	Regression coefficient (95% CI)
*Fixed effects*		

Health facility level	Log (Number of doses)	0.16 (0.072, 0.247)***
	Sub-centre as compared to primary health centre	−0.611 (−0.799, −0.424)***
	Log (distance from nearest cold chain point)	0.042 (−0.003, 0.086)
	Salary of the vaccinator (in units of US$1724)	0.156 (0.121, 0.191)***
	Proportion of DPT3 doses in total doses	−0.06 (−0.335, 0.215)
	Log (Number of sessions)	0.354 (0.229, 0.479)***

Block level	Female literacy rate	0.007 (0, 0.013)*
	% of rural population	−0.003 (−0.006, 0)*
	% of SC/ST population	0 (−0.002, 0.003)
	2nd wealth quintile	−0.111 (−0.253, 0.031)
	3rd wealth quintile	−0.156 (−0.321, 0.01)
	4th wealth quintile	−0.243 (−0.484, −0.001)*
	5th wealth quintile	−0.173 (−0.438, 0.092)

District level	Health facility per 1000 children	0.022 (−0.092, 0.136)
	% of institutional delivery	0 (−0.004, 0.004)

State-level	Log (per capita income)	0.456 (0.218, 0.695)***

Variance components	State	0.02545
	District	0.00355
	Facility	0.04235

*** Indicates p value less than 0.001.

* Indicates p value less than 0.05.

Higher female literacy rates in a block were found to be marginally associated with lower facility costs net vaccine costs. Controlling for other predictors, rural health facilities had lower costs than urban ones, and health facilities in wealthier blocks had lower costs than did those in poorer blocks. The other block- and district-level variables that we considered were not found to be significantly associated with facility cost excluding vaccine cost.

Per capita income across the study states varied widely. In 2013–14, Bihar had the lowest per capita income, at US$538, and Gujarat had the highest, at US$1842. A 25% increase in state per capita income was associated with an increase in total facility cost by US$373 (95% CI = US$112 to US$675).

The sensitivity analysis conducted using facility cost including vaccine cost as the outcome variable produced similar findings. At the facility level, the total doses administered, the type of facility (SC or PHC), the salary of the main vaccinator, and the number of immunization sessions were significantly associated with the total facility cost. The distance of a facility from the nearest cold chain point was significantly associated with costs calculated, including vaccine costs.

## 5 Discussion

This study presents the determinants of routine immunization programme costs in India using data from 99 SCs and 89 PHCs across seven states. From the cost-determinant model, we found that the main determinants of total facility costs were total doses administered; the type of facility (SC or PHC); the salary of the main vaccinator; the number of immunization sessions; and the distance of the facility from the nearest cold chain point. The studies in Benin and Ghana found doses administered as significant determinants of cost; however, salary was not significantly associated [5], [6]. The multi country study on determinants of immunization costs also found that higher health facility level was associated with higher costs probably because of differences in availability of staff and infrastructure [17]. Higher service volume was significantly associated with lower average cost while greater distance for vaccine collection was associated with higher facility cost.

Vaccine wastage rates are expected to be an important determinant of cost; however, we could not consider this variable in our regression model. We calculated the vaccine wastage rate at the vaccinator level for all antigens by subtracting doses administered from doses used, divided by doses used. Where an open-vial policy was applicable, we considered returned doses when we calculated wastage rates. Wastage rates for vaccines were calculated for 164 of the 255 vaccinators; for the remaining 91, we relied on the wastage rates used in India’s comprehensive multi-year plan for immunization (cMYP), because data were not available [9]. Hence, for some facilities, the wastage rate used was not actual. Because of the measurement error in wastage rate, we dropped this variable from our regression model. The multi-country study on determinants of immunization cost also could not include vaccine wastage rate as an explanatory variable because of data limitations [17].

We wanted to include immunization coverage as one of the explanatory variables, as we expect coverage will be a determinant of total cost. However, several sampled facilities reported more than 100% DPT3 coverage rate, indicating non-trivial measurement errors. For this reason, we dropped this variable as well. The multi-country study found a negative coefficient for coverage rate. It is expected that costs will increase with coverage as improving coverage will require more efforts for vaccinating hard-to-reach children. No firm conclusions were drawn on the reason of such negative relationship because of mismeasurement of reported coverage [17].

The present study has several limitations. First, for calculating cost per dose, we used data reported by administrators, and the quality of these data is questionable. India’s immunization costing study also noted several data reporting errors at all levels [9]. However, as there is no other source of such data, we had to rely on this source. Second, we did not consider two potentially important variables in our regression model, vaccine wastage rate and immunization coverage rate. These variables were not considered in our regression model because of missing data and poor record keeping. Further, greater completion of the vaccination schedule (proxied by the ratio of DPT3 to total doses) did not show any significant relationship to cost, despite having been found to have a strong positive relationship with cost in a pooled analysis of six countries [17]. Poor record keeping systems in India could be the reason for this. This analysis advocates for better record keeping in the Indian context. Providing more training of staff and introducing a real-time immunization registry could improve the system. Thirdly, we estimated our regression models using the log of total costs as our dependent variable. This assumes that explanatory variables have a multiplicative effect on total costs, and that errors are normally distributed on the log scale. While this approach is conventional for the type of data collected in this study [17], other functional forms may have produced different results. As the results of log models are difficult to interpret directly we calculated first differences to demonstrate the implications of the regression results. Fourthly, the primary study being a cross-sectional survey, we could only study associations between health facility and area-level characteristics and total cost. It is not possible from current analysis to make any causal inferences.

By identifying the determinants of immunization cost in India, this study may help policymakers to assess the impact of various strategies on total cost. Use of pentavalent vaccine was found to be positively associated with total facility cost including vaccine cost. Although only two out of seven states used pentavalent vaccine during the study period, currently all Indian states use pentavalent vaccine. This means that the total cost of the programme has increased significantly. Pentavalent vaccine was supported by Gavi during the study period, but that support has ended. Further, as several new and expensive vaccines are entering in the system (e.g., pneumococcal), total facility costs will increase significantly. Some of these new vaccines are still Gavi supported, but India will soon graduate from Gavi support; hence, the government needs to ensure the additional funding that will be required. Distance of a facility from the nearest cold chain point was significantly associated with facility cost. Currently, India’s immunization programme operates in 26,384 cold chain points, with an average population of 45,868 per cold chain point [18]. Increasing the number of cold chain points and taking them closer to facilities may help reduce vaccine delivery costs. Health facilities in wealthier blocks had lower delivery costs, suggesting better access in these blocks (both for beneficiaries and health staff) as compared to access in the poorest blocks. This analysis therefore suggests strategies that might improve value for money in India’s immunization services.

## Footnotes

2 In India’s rural health system, SCs are the most peripheral and the first contact point between the primary health care system and the community. Each PHC is a four- to six-bed referral unit for six SCs. CHC is a 30-bed hospital or referral unit of four PHCs with specialized services [10]. In each district, there is generally one district hospital; immunization activities are conducted in each district hospital’s post-partum unit.
