# Supplementary material for: Determinants of cost of routine immunization programme in India
Source: Vaccine. 2018 Jun 18;36(26):3836–41. doi: 10.1016/j.vaccine.2018.05.006 (PMC5999352; doi:10.1016/j.vaccine.2018.05.006)
Supplement: Supplementary data 1 [file mmc1.docx]

Table S1: Results of the regression analysis of total facility cost with vaccine cost (primary health centres and sub-centres combined)

| Variable category | Variables | Total facility cost with vaccine |
| --- | --- | --- |
| Fixed effects |  |  |
| Health facility level | log (Number of doses) | 0.24 (0.162, 0.317) *** |
|  | Health facility type | -0.505 (-0.627, -0.382) *** |
|  | log (distance from nearest cold chain point) | 0.043 (0.004, 0.082) * |
|  | Salary of the vaccinator (in units of US$1724) | 0.122 (0.091, 0.154) *** |
|  | Proportion of DPT3 doses in total doses | 0.032 (-0.223, 0.287) |
|  | log (Number of sessions) | 0.347 (0.241, 0.454) *** |
| Block level | Female literacy rate | 0.005 (0, 0.01) |
|  | % of rural population | -0.002 (-0.005, 0) |
|  | % of SC/ST population | 0 (-0.002, 0.002) |
|  | 2^nd^ wealth quintile | -0.094 (-0.22, 0.032) |
|  | 3^rd^ wealth quintile | -0.157 (-0.3, -0.014) * |
|  | 4^th^ wealth quintile | -0.218 (-0.429, -0.008) * |
|  | 5^th^ wealth quintile | -0.156 (-0.383, 0.07) |
| District level | Health facility per 1,000 children | 0.021 (-0.082, 0.123) |
|  | % of institutional delivery | 0.001 (-0.003, 0.004) |
| State-level | Use of pentavalent vaccine | 0.274 (0.057, 0.491) * |
|  | Log (per capita income) | 0.387 (0.181, 0.592) *** |
| Variance components | State | 0.00865 |
|  | District | 0.00270 |
|  | Facility | 0.03405 |

Table S2: Regression coefficients from separate regression analyses for primary health centres and sub-centres

| Variable category | Variables | Sub-centres | | Primary Health Centres | |
| --- | --- | --- | --- | --- | --- |
|  |  | Total facility cost with vaccine | Total facility cost without vaccine | Total facility cost with vaccine | Total facility cost without vaccine |
| Health facility level | log(Number of doses) | 0.198 (0.102, 0.295) *** | 0.149 (0.031, 0.266) * | 0.246 (0.134, 0.358) *** | 0.163 (0.041, 0.286) ** |
|  | log ( distance from nearest cold chain point) | -0.001 (-0.046, 0.044) | 0 (-0.051, 0.051) | 0.092 (0.027, 0.157) ** | 0.11 (0.04, 0.179) ** |
|  | Salary of the vaccinator (in units of US$1724) | 0.14 (0.103, 0.177) *** | 0.191 (0.15, 0.232) *** | 0.116 (0.068, 0.163) *** | 0.134 (0.082, 0.186) *** |
|  | Proportion of DPT3 doses in total doses | -0.094 (-0.366, 0.178) | -0.183 (-0.481, 0.116) | 0.143 (-0.286, 0.572) | 0.19 (-0.21, 0.589) |
|  | log ( Number of sessions) | 0.28 (0.166, 0.395) *** | 0.323 (0.169, 0.477) *** | 0.356 (0.199, 0.512) *** | 0.424 (0.267, 0.582) *** |
| Block level | Female literacy rate | 0.002 (-0.003, 0.006) | 0.005 (-0.002, 0.012) | 0.013 (0.007, 0.019) *** | 0.015 (0.009, 0.022) *** |
|  | % of rural population | -0.004 (-0.006, -0.001) ** | -0.002 (-0.005, 0.001) | -0.002 (-0.005, 0.002) | -0.003 (-0.006, 0) * |
|  | % of SC / ST population | 0.001 (-0.002, 0.003) | 0.002 (-0.001, 0.004) | 0 (-0.003, 0.003) | 0 (-0.003, 0.003) |
|  | 2^nd^ wealth quintile | -0.102 (-0.233, 0.029) | -0.043 (-0.202, 0.117) | -0.153 (-0.349, 0.044) | -0.216 (-0.423, -0.009) * |
|  | 3^rd^ wealth quintile | -0.139 (-0.284, 0.007) | -0.041 (-0.228, 0.146) | -0.274 (-0.484, -0.064) * | -0.361 (-0.584, -0.139) ** |
|  | 4^th^ wealth quintile | -0.139 (-0.317, 0.039) | -0.02 (-0.287, 0.246) | -0.42 (-0.708, -0.132) ** | -0.546 (-0.828, -0.264) *** |
|  | 5^th^ wealth quintile | -0.101 (-0.296, 0.094) | 0.007 (-0.289, 0.303) | -0.349 (-0.662, -0.035) * | -0.464 (-0.783, -0.145) ** |
| District level | Health facility per 1,000 children | 0.007 (-0.093, 0.107) | -0.016 (-0.142, 0.109) | -0.012 (-0.165, 0.142) | 0.004 (-0.145, 0.153) |
|  | % of institutional delivery | -0.002 (-0.005, 0.001) | -0.003 (-0.007, 0.001) | 0.002 (-0.003, 0.007) | 0.002 (-0.003, 0.008) |
| State-level | Use of pentavalent vaccine | 0.165 (0.006, 0.325) * |  | 0.278 (0.029, 0.527) * |  |
|  | Log (per capita income) | 0.316 (0.173, 0.459) *** | 0.296 (0.028, 0.563) * | 0.423 (0.192, 0.654) *** | 0.535 (0.32, 0.75) *** |

*** indicates p value less than 0.001; ** indicates p value less than 0.01; * indicates p value less than 0.05
